# Larvicidal Compounds Extracted from *Helicteres velutina* K. Schum (Sterculiaceae) Evaluated against *Aedes aegypti* L.

**DOI:** 10.3390/molecules24122315

**Published:** 2019-06-22

**Authors:** Diégina A. Fernandes, Renata P. C. Barros, Yanna C. F. Teles, Louise H. G. Oliveira, Jéssica B. Lima, Marcus T. Scotti, Fabíola C. Nunes, Adilva S. Conceição, Maria de Fátima Vanderlei de Souza

**Affiliations:** 1 Post graduation Program in Bioactive Natural and Synthetic Products, Research Institute for Drugs and Medicines (IPeFarM), Federal University of Paraíba, 58051-900 João Pessoa, PB, Brazil; diegina@ltf.ufpb.br (D.A.F.); renatabarros@ltf.ufpb.br (R.P.C.B.); mtscotti@gmail.com (M.T.S.); 2 Department of Chemistry and Physics, Agrarian Sciences Center, Federal University of Paraíba, 58397-000 Areia, PB, Brazil; yanna@cca.ufpb.br; 3 Biotechnology Center, Federal University of Paraíba, 58051-900 João Pessoa, PB, Brazil; louiseguimaraes@outlook.com (L.H.G.O.); fabiola@cbiotec.ufpb.br (F.C.N.); 4 Post graduation Program in Plant Biodiversity, Department of Education, University of the State of Bahia, 41150-000 Paulo Afonso, BA, Brazil; jessica.bl@hotmail.com (J.B.L.); adilva.souza@gmail.com (A.S.C.); 5 Post graduation in Development and Technological Innovation in Medicines, Research Institute for Drugs and Medicines (IPeFarM), Federal University of Paraíba, 58051-900 João Pessoa, PB, Brazil

**Keywords:** *Helicteres velutina*, in silico study, larvicidal activity, 7,4′-di-*O*-methyl-8-*O*-sulphate flavone, *Aedes aegypti*

## Abstract

*Helicteres velutina* K. Schum (Sterculiaceae), a member of Malvaceae *sensu lato*, is a Brazilian endemic plant that has been used by the indigenous tribe Pankarare as an insect repellent. A previous study has reported the isolation of terpenoids, flavonoids and pheophytins, in addition to the larvicidal activity of crude *H. velutina* extracts derived from the aerial components (leaves, branches/twigs, and flowers). The present study reports the biomonitoring of the effects of fractions and isolated compounds derived from *H. velutina* against *A. aegypti* fourth instar larvae. A crude ethanol extract was submitted to liquid–liquid extraction with hexane, dichloromethane, ethyl acetate and *n*-butanol to obtain their respective fractions. Larvicidal evaluations of the fractions were performed, and the hexane and dichloromethane fractions exhibited greater activities than the other fractions, with LC_50_ (50% lethal concentration) values of 3.88 and 5.80 mg/mL, respectively. The phytochemical study of these fractions resulted in the isolation and identification of 17 compounds. The molecules were subjected to a virtual screening protocol, and five molecules presented potential larvicidal activity after analyses of their applicability domains. When molecular docking was analysed, only three of these compounds showed an ability to bind with sterol carrier protein-2 (1PZ4), a protein found in the larval intestine. The compounds tiliroside and 7,4′-di-*O*-methyl-8-*O*-sulphate flavone showed in vitro larvicidal activity, with LC_50_ values of 0.275 mg/mL after 72 h and 0.182 mg/mL after 24 h of exposure, respectively. This is the first study to demonstrate the larvicidal activity of sulphated flavonoids against *A. aegypti*. Our results showed that the presence of the OSO_3_H group attached to C-8 of the flavonoid was crucial to the larvicidal activity. This research supports the traditional use of *H. velutina* as an alternative insecticide for the control of *A. aegypti*, which is a vector for severe arboviruses, such as dengue and chikungunya.

## 1. Introduction

*Aedes aegypti* (Diptera: Culicidae) is the primary vector for emerging and often neglected human-transmitted diseases, such as dengue, yellow fever and, more recently, the chikungunya and zika viruses [1]. *A. aegypti* is also a vector for Fever Valley Rift, a serious emerging zoonotic disease that affects cattle [2]. The primary method for the prevention of arbovirus spread is controlling the vector, especially during the adult and larval stages [3,4]. The non-selective ingestion of particles by larvae makes the use of larvicides based on digestive action a potential method for vector control [5].

Substances extracted from plants have been shown to display important larvicidal effects [6]. Previous studies have reported the activities of plant extracts on the immune systems of mosquitoes. These effects range from damaging the peritrophic membrane that protects intestinal cells to necrotic cell death [5]. Researchers have also reported that substances extracted from plants can affect nitric oxide production in mosquito haemocytes, which can cause death [7].

Synthetic insecticides remain the first line of defence against *A. aegypti*. However, starting in the 1950s, resistant strains of the insect began to be identified. This resistance to synthetic insecticides has aroused interest in identifying natural alternatives to synthetic insecticides, due to their characteristics of selectivity, safety and biodegradability [8,9]. 

Natural products derived from plants have been used to control *A. aegypti* through their larvicidal, insecticidal and repellent activities. The secondary metabolites produced by plants, such as essential oils, alkaloids and phenolics, have been shown to have several pharmaceutical and insecticidal properties [10]. Therefore, these compounds are targeted when evaluating the activities of vegetal extracts, fractions and isolated substances [11,12,13].

The species *Helicteres velutina* (Sterculiaceae), a member of Malvaceae *sensu lato* [14], is an endemic species from Brazil, where it is popularly known as “pitó” and has been traditionally used as insect repellent by the indigenous Pankarare tribe, from the Jeremoabo region (Bahia, Brazil) [15]. A previous phytochemical study on *H. velutina* resulted in the isolation of 17 compounds: triterpenes, flavonoids, a lignan, phaeophytins, a long-chain alcohol and a fatty acid. The crude extracts from this species have demonstrated larvicidal activity [15,16].

Based on the previously reported larvicidal potential of *H. velutina* extracts [16], here, we report here a biomonitoring study to identify which compounds in the extracts are responsible for the larvicidal activity [17,18]. A virtual, in silico screening process was performed to evaluate the activities of the previously identified substances by analysing their interactions with molecular targets. In vitro bioassays using *A. aegypti* larvae were performed to confirm the results obtained from the in silico evaluation.

## 2. Results

### 2.1. Larvicidal Activity of Helicteres velutina Fractions

Previous studies have demonstrated the larvicidal activity of crude ethanolic extracts (CEEs) derived from the stems, roots, and aerial components of *H. velutina,* with LC_50_ (50% lethal concentration) values of 138.896 mg/mL, 171.683 mg/mL and 2.983 mg/mL, respectively [15,16]. Based on these results, the extract derived from the aerial components was selected for this study. 

The aerial component extract was fractionated by liquid–liquid chromatography to obtain hexane, dichloromethane, ethyl acetate, *n*-butanol and hydroalcoholic fractions. Each fraction was submitted to biological tests to determine the fractions with the greatest larvicidal activities against *A. aegypti* larvae.

**Figure 1:**
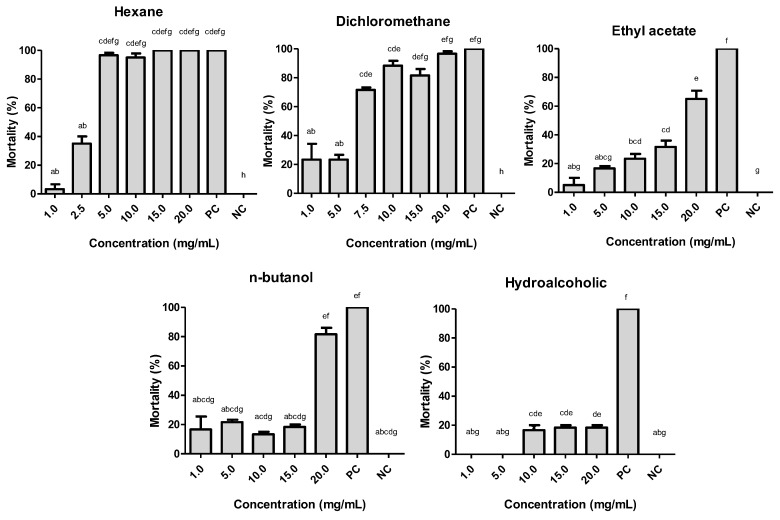
Larvicidal activity of *Helicteres velutina* fractions from crude ethanolic extract (CEE) against *A. aegypti* larvae. **PC** = positive control, **NC** = negative control. Bars with the same letter are not significantly different as determined by Tukey test, 5%.

To perform the bioassays, each fraction was used at a concentration between 1.0 and 20.0 mg/mL (Figure 1), and the LC_50_ values were determined as follows: 3.381 mg/mL for hexane; 5.801 mg/mL for dichloromethane; 13.423 mg/mL for ethyl acetate; and 10.317 mg/mL for *n*-butanol. The hydroalcoholic fraction showed an LC_50_ higher than the concentration established for the study and was therefore excluded from the study (Table S1, Supporting Information).

### 2.2. Computational Study

The identified molecules were evaluated in silico, by virtual screening, in order to select the most promising compounds to be tested against the *A. aegypti* larvae in vitro [19,20,21].

A total of 5270 molecular descriptors were calculated using the software Dragon 7.0 [22] for the bank of molecules that are active against *A. aegypti* larvae. These molecular descriptors were used to construct a prediction model, including the bank of compounds isolated from *H. velutina*. The molecular descriptors and biological activity classifications from the ChEMBL database were used as input data for the Knime software to create a prediction model using the random forest (RF) algorithm.

The quality of the model could be observed by using a confusion matrix, which reports the total correct and incorrect predictions made by the model. Table 1 summarises the results obtained from the model for training, cross-validation and testing. Training showed the best accuracy, and cross-validation and testing exhibited similar accuracy rates. The data revealed that the model had good prediction performance and robustness, with a specificity greater than 86% and a sensitivity greater than 73%.

Two parameters have been used to evaluate the quality of binary models: the receiver operating characteristic (ROC) curve and the Matthews correlation coefficient (MCC) [23].

**Figure 2:**
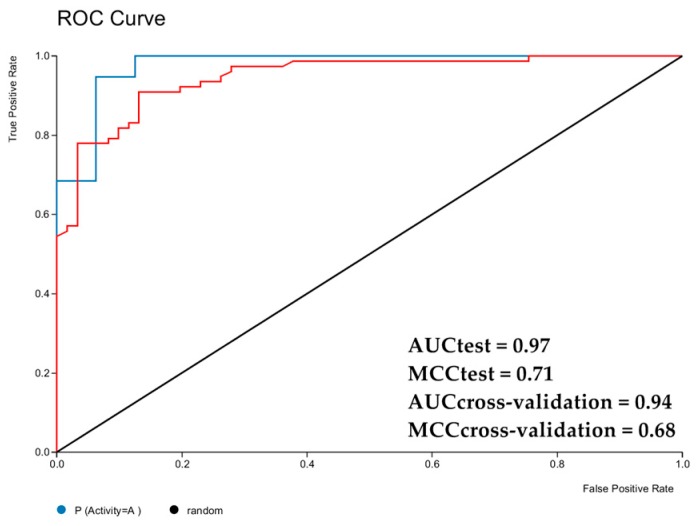
Receiver operating characteristic (ROC) plot of sensitivity versus 1-specificity, of the selected RF model for cross-validation (red line) and test sets (blue line). **AUC** = area under the curve; **MCC** = Matthews correlation coefficient.

Initially, the ROC curve is plotted to obtain an area under the sensitivity curve (true positives), which can be used to calculate 1-specificity (false positives); a value of 1 would represent a perfect model [24]. For our model, the area under the curve was greater than 94% for cross-validation and greater than 97% for the test sets, confirming that the model is able to perform with good classification and prediction rates. Figure 2 shows the testing and cross-validation ROC curves.

Likewise, the MCC parameter, which lists all confusion matrix values, was calculated using the following equation (Equation (1)):

(1) $$MCC=\frac{TP\times TN-FP\times FN}{\surd \left(TP+FP\right)\left(TP+FN\right)\left(TN+FP\right)\left(TN+FN\right)}$$

where *TP* = true positive rate, *TN* = true negative rate, *FP* = false positive rate, and *FN* = false negative rate.

An MCC value of 1 indicates a perfect correlation, 0 indicates a random prediction, and −1 indicates a total mismatch between the prediction and the observation [24]. The MCC values for testing and cross-validation in the RF models were 0.68 and 0.71, respectively, as shown in Figure 2. Fourteen molecules showed probabilities of activity greater than 50% (pIC_50_ ≥ 4.15), pIC_50_ (−log IC_50_ mol/L, noting that IC_50_ represents the minimum concentration required for a 50% inhibition of the studied microorganisms), and these compounds were selected by the model. 

**Figure 3:**
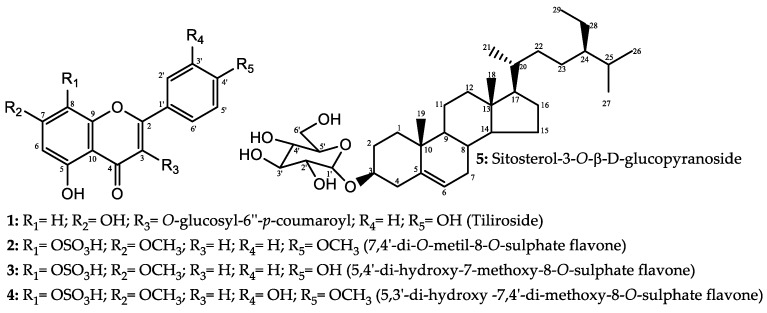
Compounds isolated from *H. velutina* considered potentially active against *A. aegypti* larvae after analysis of the applicability domain.

The applicability domain (APD) was used to assess the reliability of the predictions for the samples in the test. The APD calculation is based on molecular interactions determined by Volsurf+ descriptors. Among the fourteen selected compounds with potentially active structures (pIC_50_ ≥ 4.15), only five of them returned reliable results (Figure 3). This result indicates that several compounds were outside of the APD and were not represented among the molecules used in the training data set for the prediction model [25]. 

For the molecular docking analysis, three *A. aegypti* target proteins were selected from the Protein Data Bank (PDB): 1YIY [26], 1PZ4 [27] and 3UQI [28]. These results suggest three possible action mechanisms through which the active molecules can interact with the vector. Information regarding each protein and its respective ligands is shown in Table 2.

The molecular docking predictions were validated by redocking the original ligand with the target A. aegypti proteins. Table 3 shows their MolDock scores, the root mean square deviation (RMSD) values and the energies from the PDB. 

A virtual screening protocol, using the same parameters for all proteins, was performed on the reliable isolated compounds. Based on the binding energy values, the tested molecules were ranked using the following probability calculation (Equation (2)):

(2) $$ps=\frac{E_{TM}}{E_{M}},\;IF\;E_{TM}<E_{L}$$

where *ps* = structure-based probability; *E_TM_* = test molecule docking energy and TM ranges from 1 to 1848 (secondary metabolites dataset); *E_M_* = lowest energy value obtained from the tested molecules, which, for the three proteins examined here, was tiliroside, which returned energy values of −163.330, −188.175 and −154.188 kJ/mol for 1YIY, 1PZ4, and 3UQI, respectively; and *E_L_* = ligand energy with crystallographic protein. 

Equation (2) aims to normalise the scores from molecular docking (structure-based virtual screening), allowing the values from the structure-based virtual screening to be compared with the values from the ligand-based virtual screening. The selection of the structures was based on their energies. The energy must be lower than the value obtained for the ligand in the crystallography study. The investigated molecules were classified as active if the structure-based probability values were greater than or equal to 0.5.

The number of molecules identified with probability values greater than 0.5 and binding energy values lower than that for the binder were as follows: eight for protein 1YIY, eight for protein 1PZ4 and nine for protein 3UQI.

**Figure 4:**
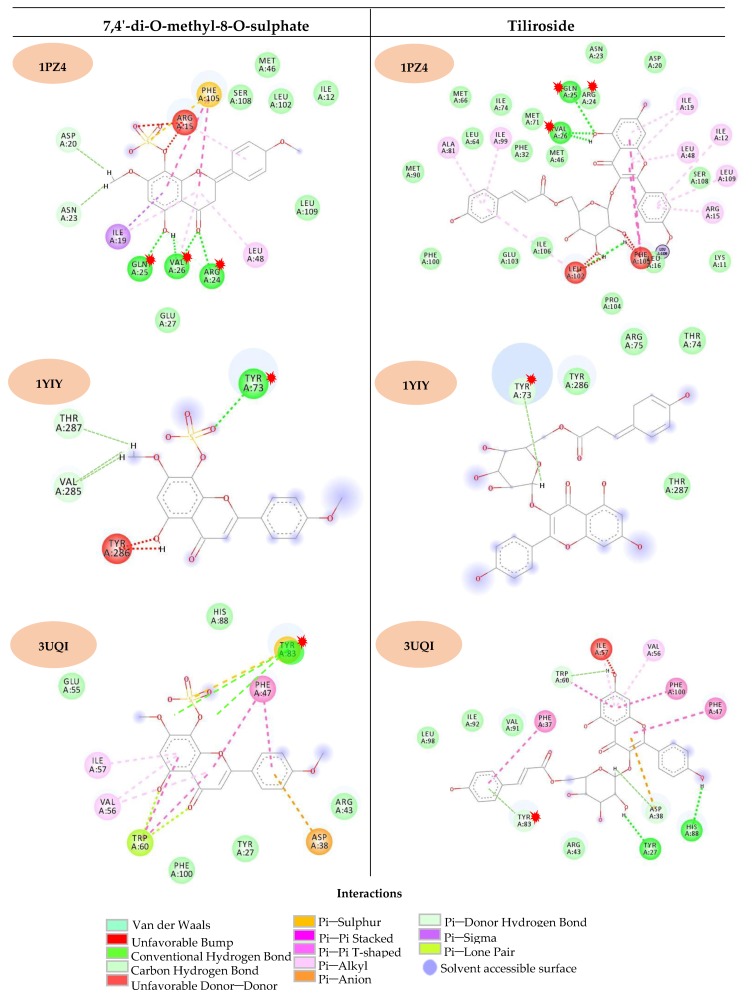
Interactions of the compounds tilirosideo and 7,4′-di-*O*-methyl-8-*O*-sulphate flavone with the proteins active sites. The amino acids residues in common for the two substances are marked with a red asterisk and represent the hydrogen interactions.

An evaluation using structure-based and ligand-based virtual screening was performed to verify potentially active molecules and their possible mechanisms of action, showing potential multitarget molecules. This approach also seeks to minimise the probability of selecting false positive molecules because it considers the scores of virtual tracking techniques and correlates them with the true negative rate [29,30]. The calculation was performed using the following equation:

(3) $$Pc=\frac{ps+\left(1+TN\right)\times p}{2+TN},\;IF\;Pc>0.5$$

where *Pc* is the combined probability; *ps* is the structure-based probability, *TN* is the true negative rate, and *p* is the ligand-based probability. In this equation, the ligand-based score is correlated with a decreasing false positive rate with incremental increases in the *TN* value. Thus, the probability of incorrectly identifying inactive molecules as active molecules is minimised. Table 4 shows the results for the best-ranked molecules obtained using this combined approach, and Figure 4 shows the best-classified structures.

The five substances that showed potential activity against *A. aegypti* in the prediction model also showed good activity in the molecular docking study. The best results were attributed to tiliroside, 7,4′-di-*O*-methyl-8-*O*-sulphate flavone and sitosterol-3-*O*-β-d-glucopyranoside, with good probabilities of activity against all three proteins, especially against 1PZ4, which is found in the larval intestine. Reports of the larvicidal activity of sitosterol-3-*O*-β-d-glucopyranoside were found in the literature [31]; thus, further studies were performed using tiliroside and 7,4′-di-*O*-methyl-8-*O*-sulphate flavone.

Figure 4 shows the interactions between the protein active sites and tiliroside and 7,4′-di-*O*-methyl-8-*O*-sulphate flavone, highlighting the amino acids residues in common between the two compounds.

No conclusions can be formed regarding the compounds that were not tested in vitro. The virtual screening was performed with the intention of selecting promising compounds for in vitro testing to avoid wasting materials and to minimise costs.

### 2.3. Larvicidal Activity of the Promising Substances

**Figure 5:**
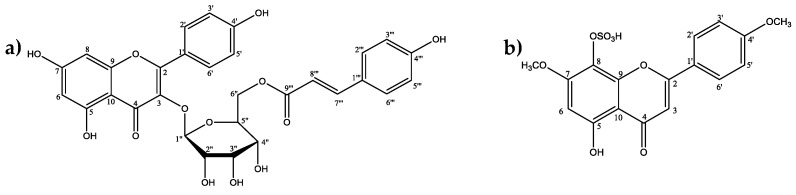
Substances selected after virtual screening for in vitro testing: (**a**) Tiliroside; (**b**) 7,4′-di-*O*-methyl-8-*O*-sulphate flavone.

From the virtual screening results, two flavonoids with potential larvicidal activities were selected: tiliroside and 7,4′-di-*O*-methyl-8-*O*-sulphate flavone (Figure 5). These compounds, isolated from *H. velutina*, were submitted to in vitro tests to confirm their larvicidal activities.

**Figure 6:**
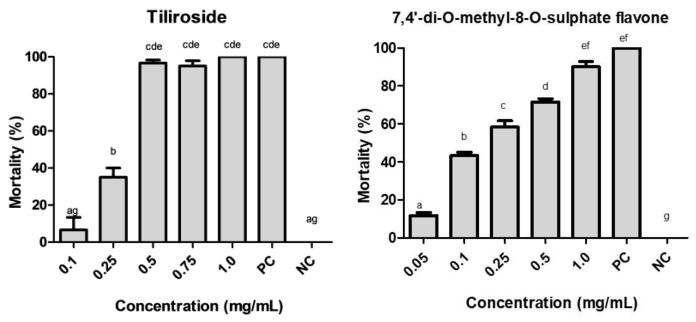
Larvicidal activity of the isolated substances of *H. velutina,* tiliroside after 72 h and 7,4′-di-*O*-methyl-8-*O*-sulphate flavone after 24 h. **PC** = positive control, **NC** = negative control. Bars with the same letter are not significantly different as determined by Tukey test, 5%.

The average mortality of *A. aegypti* larvae exposed to different concentrations of tiliroside and 7,4′-di-*O*-methyl-8-*O*-sulphate flavone was determined (Table S2, Supporting Information). At a concentration of 1.0 mg/mL, it was possible to observe significant larvicidal activity for both substances. 7,4′-Di-*O*-methyl-8-*O*-sulphate flavone resulted in a larval mortality of 90% after 24 h (Figure 6), reaching 100% after 48 h. Tiliroside killed all larvae at the same concentration (1.0 mg/mL) within 72 h (Figure 6). The calculated LC_50_ values were 0.182 and 0.275 mg/mL, respectively, for tiliroside and 7,4′-di-*O*-methyl-8-*O*-sulphate flavone.

The in silico evaluation showed an interesting result for the compounds 7,4′-di-*O*-methyl isoscutellarein and 7,4′-di-*O*-methyl-8-*O*-sulphate flavone. These structures differ from each other by the presence of a sulphate group (OSO_3_H) at the C-8 position of the flavonoid nucleus of sulphated isoscutellarein; however, only 7,4′-di-*O*-methyl-8-*O*-sulphate flavone was predicted to be active. To confirm the results from the in silico evaluation, in vitro tests were performed using 7,4′-di-*O*-methyl isoscutellarein and 7,4′-di-*O*-methyl-8-*O*-sulphate flavone. A concentration of 1.0 mg/mL was used for both compounds. After 72 h of exposure, 7,4′-di-*O*-methyl isoscutellarein presented 21.6% larval mortality (Table S2, Supporting Information), while the 7,4′-di-*O*-methyl-8-*O*-sulphate flavone presented 100% larvicidal activity. These in vitro results confirmed the results of the computational study, suggesting that the *O*-sulphate group at the C-8 position is associated with the strong larvicidal activity observed for 7,4′-di-*O*-methyl-8-*O*-sulphate flavone.

## 3. Discussion

The current absence of effective vaccines against arboviruses transmitted by *A. aegypti* have led scientists to focus on controlling the vector [3,10]. Natural products have traditionally been used by local populations against several species of insects [15,32]. Many plant species have displayed efficacies as larvicides and adulticides against *A. aegypti* [8,11,33]. Bioactive compounds isolated from plants are biodegradable and environmentally friendly, with specific actions that are considered to be non-toxic to other insects [4,34]. Previous studies have reported the promising efficacies of the natural biological insecticides azadirachtin, pyrethrins, rotenone, nicotine and toosendanin [11,35,36]. 

Biomonitored phytochemical research aims to identify which substances are responsible for specific biological activities [37,38,39]. Biomonitored research involves studies across several knowledge areas to identify the bioactive compounds of interest [40].

The *H. velutina* CEE was partitioned into hexane, dichloromethane, ethyl acetate, *n*-butanol and hydroalcoholic fractions. Of these five fractions, only the hexane and dichloromethane fractions presented promising activities against *A. aegypti* larvae, with LC_50_ values of 3.881 mg/mL and 5.801 mg/mL, respectively. These LC_50_ values are larger than that recorded for CEE (LC_50_ 2.983 mg/mL), characterizing the natural synergism of the crude extract [41,42,43]. The higher polarity fractions (ethyl acetate, *n*-butanolic and hydroalcoholic fractions) only demonstrated larvicidal activities when using significantly higher concentrations, presenting unsatisfactory results.

The observed results were similar to those described in previous studies. A study performed using a hexane extract of *Zanthoxylum oxyphyllum* leaves showed larvicidal activity with an LC_50_ value of 5.990 mg/mL [44]. The hexane extract of *Murraya koeniggi* leaves also showed good activity against *A. aegypti*, followed by the chloroform and methanolic extracts [45]. The hexane fraction of *Murraya koeniggi* leaves was shown to be the most promising fraction, with larvicidal activity at a concentration of 0.035 mg/mL [43,46]. Biological activity at low concentrations is the result of very active components within the fractions [43,46]. Other studies have also reported better activities for the hexane and dichloromethane fractions [3,35,47].

These data show that larvicidal activity can vary depending on the solvent used for extraction or partitioning because each solvent has the ability to selectively extract a different group of constituents according to polarity [18]. Ethanol, methanol, hexane and chloroform are the solvents that are generally used to extract and partition natural products [48,49,50]. The extracts and fractions obtained from plant species can exert their toxic effects on larvae through multiple methods, such as the inhibition of growth, reproduction, or fertility [51].

The *H. velutina* phytochemical study described here was developed based on the biological results of the hexane and dichloromethane fractions. Using chromatographic procedures, 17 substances were identified, five of which were isolated for the first time from the genus *Helicteres*, and two new compounds with larvicidal activity were identified [16].

The molecules were subjected to a virtual screening protocol to select those that were most likely to be active against *A. aegypti* [19,23,24]. The molecules with a greater than 50% probability of being active (14 compounds) were selected by the model. The substances 7,4′-di-*O*-methyl isoscutellarein, kaempferol and palmitic acid were considered to be inactive. 

After the APD analysis, only five compounds were classified as reliable. The other molecules were classified as unreliable, which can be attributed to the fact that the dataset of the model did not contain similar structures for larvicidal activity against *A. aegypti* as those described in the literature [25].

Molecular docking was validated for the three studied proteins, with low RMSD values upon redocking. By analysing the MolDock energy values of each studied enzyme, the molecules that were likely to interact with the active site of the enzymes were identified. Tiliroside, glucosylated β-sitosterol and 7,4′-di-*O*-methyl-8-*O*-sulphate flavone were identified as having high probabilities of interacting with the three analysed proteins (1YIY, 1PZ4 and 3UQI), illustrating that these compounds could act on the vector through different mechanisms. Other molecules with interesting results included mariahine and condadine, which presented the ability to dock with two enzymes. 

As shown in Figure 4, we observed the common critical amino acid residues in tiliroside and the 7,4′-di-*O*-methyl-8-*O*-sulphate flavone that interact with active sites of the proteins. Both tested compounds interact with the active sites through hydrogen bonds with oxygenated species, primarily the phenolic hydroxyls and the sulphate group of 7,4′-di-*O*-methyl-8-*O*-sulphate flavone. For the 1YIY enzyme, only the serine 252 residue interacts with both compounds; however, for 7,4′-di-*O*-methyl-8-*O*-sulphate flavone, three other residues interact by forming hydrogen bonds with the sulphated group. This trend was also observed for the other proteins, and the sulphate group appears to be responsible for several interactions with the active-site amino acid residues, which may result in their high probability of being active in the docking study.

Our findings showed that the best in silico results were exhibited for flavonoids and steroids. These compounds possess certain characteristics, such as planar and aromatic rings, sulphate groups, and sugars, which may be crucial to their biological activities [19].

Flavonoids are known for their many pharmacological functions. Structural changes, such as sulphation, methylation and glycosylation, usually change their solubility, stability and biological activities [52]. The antiviral and anticoagulant activities of sulphated flavonoids have been well-described [53]. Tiliroside has been shown to have cytotoxic, hepatoprotective, vasorelaxative, hypoglycaemic, anti-inflammatory, and antioxidant activities [54,55,56,57,58,59]. β-Sitosterol, both in its free and glycosylated forms, has been shown to have anti-inflammatory and larvicidal activities against *A. aegypti* [31,60].

The compounds tiliroside and 7,4′-di-*O*-methyl-8-*O*-sulphate flavone were considered to be promising compounds and were selected for evaluation using an in vitro assay. According to the Tukey test, the larvicidal activities of tiliroside did not differ significantly among concentrations of 0.5 mg/mL, 0.75 mg/mL and 1.0 mg/mL after 72 h of exposure. For 7,4′-di-*O*-methyl-8-*O*-sulphate flavone, the larvicidal activities did not differ significantly among concentrations of 0.1 mg/mL, 0.25 mg/mL, 0.5 mg/mL and 1.0 mg/mL after 24 h of exposure. The LC_50_ values calculated for tiliroside and 7,4′-di-*O*-methyl-8-*O*-sulphate flavone were 0.275 mg/mL and 0.182 mg/mL, respectively, showing that both compounds were active during different time intervals.

This is the first report of larvicidal activity for tilirosideo and 7,4′-di-*O*-methyl-8-*O*-sulphate flavone against *A. aegypti*. Other flavonoids, such as poncirin, rhoifolin, naringin and marmesin, present similar levels of activity, with LC_50_ values ranging from 0.082 to 0.122 mg/L after 24 h of exposure [61]. The compounds karanjin, karanjachromene, the dihydrochalcone flavonoid pongamol and the rotenoid flavonoid pongarotene exhibithed LC_50_ values between 0.0161 and 0.0376 mg/mL after 24 h of exposure [62]. Other studies have investigated the larvicidal potential of flavonoids against *A. aegypti* [63,64].

This is the first study showing the activity of sulphated flavonoids against *A. aegypti* [52]. The in vitro test comparing 7,4′-di-*O*-methyl isoscutellarein and 7,4′-di-*O*-methyl-8-*O*-sulphate flavone showed that the presence of the OSO_3_H group attached to the C-8 of the flavonoid was crucial to the observed larvicidal activity. 

## 4. Materials and Methods

### 4.1. Collection, Extraction, and Compound Isolation

The procedures of collection, identification, extraction, preparation of fractions and isolation of compounds used here have been previously reported [16]. As mentioned, the hexane and dichloromethane fractions were chromatographed to isolate the compounds. The obtained compounds have been identified by spectroscopic methods [16].

### 4.2. Virtual Screening Focused on Medicinal Chemistry

From the ChEMBL database, 161 structures were selected, including secondary metabolites related to larvicidal activity (in vitro) against *A. aegypti.* The compounds were classified as active (85) (pIC_50_ ≥ 4.15) or inactive (76) (pIC_50_ < 4.15). After a literature search, 11 flavonoids and palmitic acid were added. They had known activity against *A. aegypti* larvae, with six being identified as active and six as inactive, based on the cutoff point (pIC_50_). This survey was used to maximise the representativity of the chemical space for each class of structure and to reduce the false positive rate for the model. Then, our database was built with 173 molecules. In parallel, another bank was built with the isolated substances from *H. velutina*. 

These databases were used during the virtual screening procedure to select those molecules with greater probabilities to be active against *A. aegypti*. For all structures, the SMILES codes were used as the input data in Marvin 18.10.0, 2018, ChemAxon (www.chemaxon.com). We used Standardizer software (Chem 17.29.0, 2017; ChemAxon (www.chemaxon.com)) to standardise the structures. To generate the predictive model, Knime software 3.4.0 (Knime 3.4.0, Copyright Miner Konstanz Information, 2003–2014, www.knime.org) was used. Descriptors were selected, and a model was generated using the training set and the random forest algorithm (RF), using Waikato Environment for Knowledge Analysis (WEKA) nodes. 

The internal and external performances of the selected models have been analysed for sensitivity (true positive rate, i.e., active rate), specificity (true negative rate, i.e., inactive rate), and accuracy (overall predictability). To describe the true performance of the model with more clarity than accuracy, the sensitivity and specificity of the ROC were determined.

APD analysis was used to identify the compounds within the test sets and to evaluate whether the predictions were reliable. The APD is based on Euclidean distances and similarity measures among the descriptors of the training set. If the compounds in the set tests have distances and similarities beyond this limit, then the predictions of the model are not reliable. 

### 4.3. Molecular Docking

The structures of three *A. aegypti* proteins (1YIY [25], 1PZ4 [26] and 3UQI [27]), complexed with their inhibitors, were downloaded from the Protein Data Bank (PDB), with each protein referring to an *A. aegypti* action mechanism. The water molecules were eliminated from the enzyme structures. The structures of the proteins and of the compounds were prepared using the same standard parameters in Molegro Virtual Docker (v 6.0.1, Molegro ApS, Aarhus, Denmark).

### 4.4. Larvicidal Activity

The larvicidal activity of *H. velutina* fractions and isolated substances were evaluated according to the World Health Organization recommendations (1970) [65]. *A. aegypti* larvae in the fourth stage (L4) (Cepa Rockefeller, João Pessoa) were obtained from Laboratório de Biotecnologia Aplicada a Parasitas e Vetores (Centro de Biotecnologia, Universidade Federal da Paraíba). 

The test substances were diluted in water or water containing 1% DMSO, at different concentrations. Twenty fourth-stage larvae were transferred into Falcon tubes containing the test solutions. The negative control group was prepared with water or water containing 1% DMSO. The positive control group was prepared with a commercial insecticide: imiprothrin 0.2 mg/mL, permethrin 0.5 mg/mL and esbiothrin 1.0 mg/mL. 

The tubes were examined after 24 h for the fractions and after 24 to 72 h for the isolated compounds. They were kept under conditions of biological oxygen demand (BOD), at 27 ± 2 °C, relative humidity of 27 ± 5 °C, and a photoperiod of 12 h light and 12 h dark. All tests were performed in triplicate. 

The results were evaluated using GraphPad Prism (v 5.0, GraphPad Software, San Diego, CA, USA). Analysis of variance (ANOVA) and Tukey’s post hoc test (*P* < 0.05) were applied to determine the significant differences between groups. 

## 5. Conclusions

The biomonitored, phytochemical study of extracts derived from the aerial components of *H. velutina* against *A. aegypti* larvae resulted in the discovery of the promising biological activities of the hexane and dichloromethane fractions. From those fractions, chromatographic and spectroscopic methods resulted in the isolation and identification of 17 substances. The compounds were submitted to an *in silico* study to select those with greater probabilities of demonstrating larvicidal activities against *A. aegypti*. Three compounds were found to be able to bind the 1PZ4 protein, found in the *A. aegypti* larval intestine. Two isolated flavonoids (tiliroside and 7,4′-di-*O*-methyl-8-*O*-sulphate flavone) were submitted to in vitro assays, which demonstrated that these compounds showed larvicidal activities at low concentrations. 

This is the first study to demonstrate the activity of sulphated flavonoids against *A. aegypti*. Our results showed that the presence of the OSO_3_H group attached to C-8 of the flavonoid was crucial to its larvicidal activity. This research supports the traditional use of *H. velutina* as an alternative insecticide for the control of *A. aegypti*, which is a vector of severe arboviruses, such as dengue and chikungunya.

## Figures and Tables

**Table 1:** Summary of integral training, cross-validation and testing with the corresponding match results using the random forest (RF) algorithm.

	Training			Cross Validation			Test		
	Samples	Matches	% Matches	Samples	Matches	% Matches	Samples	Matches	% Matches
Active	77	76	98%	77	65	84%	19	14	73%
Inactive	61	61	100%	61	53	86%	16	15	93%
Total	138	137	99%	138	118	85%	35	29	82%

**Table 2:** Information about the target proteins of *A. aegypti* and their respective ligand.

Protein ID	Classification	Ligand	Localisation
*Aedes aegypti* kynurenine aminotransferase (1YIY)	Transferase	4′-Deoxy-4′-aminopyridoxal-5′-phosphate; Pyridoxamine-5′-phosphate	Adult mosquito head
Sterol Carrier Protein-2 (1PZ4)	Lipid binding	Palmitic acid	Large intestine of larvae
AaFKBP12 (3UQI)	Isomerase	3 [*N*-morpholino]propane sulphonic acid	Adult mosquito

**Table 3:** The docking energy (kJ/mol) of the ligand for each protein from the Protein Data Bank (PDB), ligand energy of the MolDock score and the RMSD values obtained from the redocking procedure.

Protein	Energy, PDB Value (kJ/mol)	Energy, MolDock Value (kJ/mol)	Redocking, RMSD Value
1YIY	−103.002	−124.274	0.158
1PZ4	−106.678	−110.680	0.203
3UQI	−54.802	−57.032	0.138

**Table 4:** Summary of the best-ranked structures obtained using an approach combining ligand-based and structure-based virtual screening (VS); p = active probability value in ligand-based VS; ps = active probability value in structure-based VS; Pc = combined probability value.

Molecule	Protein	p	ps	Pc
Tiliroside	1YIY	0.79	1	0.79
	1PZ4	0.79	1	0.79
	3UQI	0.79	1	0.79
7,4′-di-*O*-methyl-8-*O*-sulphate flavone	1YIY	0.66	0.81	0.73
	1PZ4	0.66	0.575	0.63
	3UQI	0.66	0.45	0.59
5,4′–di-hydroxy-7-methoxy-8-*O*-sulphate flavone (mariahine)	1YIY	0.64	0.83	0.60
	1PZ4	0.64	0	0.41
	3UQI	0.64	0.53	0.53
5,3′–di-hydroxy-7,4′-dimethoxy-8-*O*-sulphate flavone (condadine)	1YIY	0.70	0.86	0.66
	1PZ4	0.70	0	0.45
	3UQI	0.70	0.38	0.54
Sitosterol-3-*O*-β-d-glucopyranoside	1YIY	0.71	0.91	0.68
	1PZ4	0.71	0.76	0.65
	3UQI	10.71	0.64	0.62

## Supplementary Materials

The following are available online. Table S1, Mean of mortality of *A. aegypti* larvae (L4) in the tested concentrations of CEE fractions of *H. velutina*, Table S2, Mean of mortality of *A. aegypti* larvae (L4) in different concentrations of tested compounds.
