# Supplementary material for: Larvicidal Compounds Extracted from Helicteres velutina K. Schum (Sterculiaceae) Evaluated against Aedes aegypti L
Source: Molecules. 2019 Jun 22;24(12):2315. doi: 10.3390/molecules24122315 (PMC6631904; doi:10.3390/molecules24122315)
Supplement: Supplementary file 1 [file molecules-24-02315-s001.pdf]

Supporting information

Larvicidal compounds extracted from *Helicteres velutina* K. Schum (Sterculiaceae) evaluated against *Aedes aegypti* L.

Diégina A. Fernandes [1], Renata P. C. Barros [1], Yanna C. F. Teles [2], Louise H. G. Oliveira [3], Jéssica B. Lima [4], Marcus T. Scotti [1], Fabíola C. Nunes [3], Adilva S. Conceição [4] and Maria de Fátima Vanderlei de Souza [1,5,*]

[1] Post graduation Program in Bioactive Natural and Synthetic Products; Federal University of Paraíba, 58051-900, João Pessoa, PB, Brazil; diegina@ltf.ufpb.br (D.A.F.); renatabarros@ltf.ufpb.br (R.P.C.B.); mtscotti@gmail.com (M.T.S.); mfvanderlei@ltf.ufpb.br (M.F.V.S.)

[2] Department of Chemistry and Physics, Agrarian Sciences Center, Federal University of Paraíba, Areia, PB, Brazil; yanna@cca.ufpb.br (Y.C.F.T.)

[3] Biotechnology Center; Federal University of Paraíba, 58051-900, João Pessoa, PB, Brazil; louiseguimaraes@outlook.com (L.H.G.O.); fabiola@cbiotec.ufpb.br (F.C.N.)

[4] Post graduation Program in Plant Biodiversity; University of the State of Bahia; Department of Education, 41150-000, Paulo Afonso, BA, Brazil; jessica.bl@hotmail.com (J.B.L); adilva.souza@gmail.com (A.S.C.)

[5] Post graduation in Development and Technological Innovation in Medicines; Federal University of Paraíba, 58051-900, João Pessoa, PB, Brazil; mfvanderlei@ltf.ufpb.br (M.F.V.S.)

* Correspondence: mfvanderlei@ltf.ufpb.br; Tel.: +55-83-3216-7351; Fax: +55-83-3216-7351

Table S1. Mean of mortality of *A. aegypti* larvae (L4) in the tested concentrations of CEE fractions of *H. velutina*

Fractions	Concentration (mg/mL)	% Mortality ± Standard Deviation	Fractions	Concentration (mg/mL)	% Mortality ± Standard Deviation
Hexane	1.0 [(a)*]	3.0 ± 0.66	n-butanol	1.0 [(h)]	13.0 ± 1.76
	2.5	35.0 ± 1.00		5.0 [(h)]	15.0 ± 1.00
	5.0	96.5 ± 0.33		10.0 [(h)]	16.5 ± 0.88
	10.0	95.0 ± 0.57		15.0 [(h)]	11.5 ± 0.33
	15.0	100.0 ± 0		20.0	81.5 ± 0.88
	20.0	100.0 ± 0	Hydroalcoholic	1.0 [(i)]	0 ± 0
Dichloromethane	1.0 [(b)]	23.3 ± 2.18		5.0 [(i)]	0 ± 0
	5.0 [(b)]	23.3 ± 0.66		10.0 [(j)]	1.6 ± 0.57
	7.5 [(c)]	71.5 ± 0.33		15.0 [(i)]	5.0 ± 1.00
	10.0 [(c) (d)]	88.0 ± 0.66		20.0 [(j)]	11.5 ± 0.33
	15.0 [(c) (d)]	81.5 ± 0.88	Negative Control	H_2O or H_2O + DMSO 1% [(a) (e) (f) (h) (i)]	0 ± 0
	20.0 [(d)]	96.5 ± 0.33	Positive Control	Commercial larvicide	100.0 ± 0
Ethyl acetate	1.0 [(e)]	5.0 ± 1.00			
	5.0 [(e) (f)]	13.0 ± 0.33			
	10.0 [(f) (g)]	23.0 ± 0.66			
	15.0 [(g)]	31.5 ± 0.88			
	20.0	65.0 ± 1.15			

(*) Means followed by the same letter are not significantly different by Tukey test, at 5% of probability.

Table S2. Mean of mortality of *A. aegypti* larvae (L4) in different concentrations of tested compounds

Molecule	Concentration (mg/mL)	% Mortality ± Standard Deviation (Triplicate)		
		24h	48h	72h
Tiliroside	0.1 [(a)*]	0.0 ± 0.5	21.6 ± 1.3	46.6 ± 0.5
	0.25	3.3 ± 0.5	43.3 ± 0.5	70.0 ± 1.0
	0.50 [(b) (c)]	6.6 ± 1.1	48.3 ± 0.5	83.3 ± 0.5
	0.75 [(c) (d)]	11.6 ± 0.5	60.0 ± 1.0	100.0 ± 1.0
	1.0 [(b) (d) (e)]	15.0 ± 1.0	60.0 ± 1.0	100.0 ± 1.0
7,4′-di-O-methyl-8-O-sulphate flavone	0.05	11.6 ± 0.5	20,0 ± 1,0	20,0 ± 1,0
	0.1	43.3 ± 0.5	48.3 ± 1.3	51.6 ± 0.5
	0.25	58.3 ± 1.1	88.3 ± 1.5	100.0 ± 1.0
	0.50	71.6 ± 0.5	93.3 ± 1.5	100.0 ± 1.0
	1.0 [(f)]	90.0 ± 1.0	100.0 ± 1.0	100.0 ± 1.0
7,4′-di-O-methyl isoscutellarein	1.0	21.6 ± 1.5	21.6 ± 1.5	21.6 ± 1.5
Negative Control	H_2O + DMSO 1% [(a) (e)]	0.0 ± 0	0.0 ± 0	0.0 ± 0
Positive Control	Commercial larvicide [(b) (c) (f)]	100.0 ± 0	-	-

(*) Means followed by the same letter are not significantly different by Tukey test, at a level of 5% of probability.
